# Anti-ANGPTL3 antibody and IL-22 fusion protein mitigate nephrotic syndrome via mitochondrial protection, anti-apoptosis, and autophagy suppression

**DOI:** 10.1093/abt/tbag031

**Published:** 2026-06-16

**Authors:** Qianqian Ma, Leilin Shao, Guangjun Jing, Fangyu Liu, Kaicheng Zhou, Fujie Wen, Haidong He, Dianwen Ju, Hong Xu

**Affiliations:** Neonatal Intensive Care Uite, Shandong Medical and Pharmaceutical University Hospital, Binzhou 256600, China; Department of Nephrology, Children’s Hospital of Fudan University, National Children’s Medical Center, Shanghai 201102, China; Department of Nephrology, Children’s Hospital of Fudan University, National Children’s Medical Center, Shanghai 201102, China; iCareab Biotechnology Co. Ltd, Suzhou 215000, China; Department of Neurology, Shandong Medical and Pharmaceutical University Hospital, Binzhou 256600, China; Department of Biological Medicines & Shanghai Engineering Research Center of Immunotherapeutics, School of Pharmacy, Fudan University, Shanghai 201203, China; First People's Hospital of Kashi No. 120, Xinjiang 844000, China; Department of Nephrology, Minhang Hospital, Fudan University, Shanghai 201199, China; Department of Biological Medicines & Shanghai Engineering Research Center of Immunotherapeutics, School of Pharmacy, Fudan University, Shanghai 201203, China; Department of Nephrology, Children’s Hospital of Fudan University, National Children’s Medical Center, Shanghai 201102, China; Greater Bay Area Institute of Precision Medicine, Guangzhou 510000, China

**Keywords:** nephrotic syndrome, angiopoietin-like protein 3, interleukin-22, bifunctional fusion protein, mitochondria, apoptosis, autophagy

## Abstract

**Backgroud:**

Nephrotic syndrome (NS) is a major cause of end-stage renal disease. Treating NS relies on immunosuppressants, which have numerous side effects. Therefore, there is an urgent need to identify effective and safe alternative treatments for NS. Angiopoietin-like protein 3 (ANGPTL3) exacerbates proteinuria, whereas interleukin (IL)-22 has a reparative effect on renal cells.

**Methods:**

In the present study, we developed a bifunctional anti-ANGPTL3/IL-22 fusion protein and validated its efficacy in an adriamycin-induced nephropathy in mice.

**Results:**

The fusion protein significantly decreased the urinary albumin-to-creatinine ratio, serum creatinine, blood urea nitrogen, and total cholesterol levels while increasing serum albumin levels. Pathological renal damage was also alleviated. These therapeutic effects were accompanied by the preservation of mitochondrial integrity, reduced apoptosis, and inhibited autophagy. Finally, we humanized the fusion protein to facilitate its potential clinical translation.

**Conclusions:**

In conclusion, our results revealed that the anti-ANGPTL3/IL-22 bifunctional fusion protein ameliorates NS by protecting mitochondria, inhibiting apoptosis, and suppressing autophagy, highlighting a novel therapeutic approach for NS.

## Teaser

**Statement of Significance** This study applies a novel anti-ANGPTL3/IL-22 bifunctional protein to nephrotic syndrome, demonstrating its efficacy in mitigating oxidative stress, apoptosis, and autophagy in mice. Subsequent humanization confirms its translational potential as a targeted renal therapy.

## Introduction

Nephrotic syndrome (NS), characterized by massive proteinuria, hypoproteinemia, edema, and hyperlipidemia, is considered one of the leading causes of end-stage renal disease (ESRD) [1]. Although its etiology is unknown, the pathogenesis of NS is thought to be related to immune dysregulation, systemic circulatory factors, and structural abnormalities in podocytes [2]. Immunosuppressive therapy is the most common treatment option for NS; however, concerns regarding the side effects of immunosuppressants and immune resistance remain significant. Patients rely on renal replacement therapy after NS progresses to ESRD, placing a significant financial burden on both individuals and their families. Therefore, new NS treatments that delay disease progression are urgently needed.

Podocyte injury is closely related to proteinuria and is a central aspect of NS development. Autophagy is a pivotal self-stabilizing mechanism that plays an important role in maintaining podocyte integrity [3]. Disordered autophagy can strongly interfere with normal cell physiology, resulting in ischemia and reperfusion-induced damage, calcium ion homeostasis imbalance, reactive oxygen species (ROS) accumulation, and eventually apoptosis and shedding [4]. In an animal model of adriamycin-induced glomerulonephritis, when autophagy in podocytes was alleviated, abnormal mitochondria, increased ROS production, and higher apoptosis rates were observed, along with significantly aggravated glomerulosclerosis and proteinuria [5]. Recent studies have suggested that podocyte injury occurs in various renal diseases involving cellular autophagy, which can contribute to the subsequent increase in oxidative stress, mitochondrial dysfunction, and apoptosis. Therefore, patients with NS may benefit from therapeutic strategies that restore normal autophagy.

Angiopoietin-like protein 3 (ANGPTL3) is a multifunctional secreted protein predominantly expressed in the liver. It has an N-terminal coiled-coil domain (CCD) and a C-terminal fibrinogen-like domain (FLD). The ANGPTL3-FLD domain plays a key role in rearranging the podocyte cytoskeleton and inducing cell adhesion loss [6]. Previously, our group first demonstrated that ANGPTL3 is involved in podocyte injury and proteinuria, identifying it as a key mediator in these processes. Immunofluorescence and co-immunoprecipitation assays confirmed the co-localization and direct interaction between ANGPTL3 and integrin αVβ3 on injured podocytes [7]. This interaction activates the integrin αVβ3/FAK/PI3K signaling pathway, leading to podocyte cytoskeletal rearrangement and increased migration [7, 8]. Treatment with recombinant ANGPTL3 protein downregulated the expression of integrin α3β1 on the podocyte surface while upregulating Integrin-Linked Kinase (ILK) and phosphorylated integrin β1, resulting in increased podocyte detachment. Angptl3 knockout decreased urinary protein by ~80% in the adriamycin-induced nephropathy (ADRN) model [7]. In addition, further experiments confirmed that intervention with the anti ANGPTL3 FLD monoclonal antibody significantly reduced urinary protein levels and podocyte injury in mice with ADRN, while also ameliorating hypoalbuminemia and hyperlipidemia in the model mice [9].

Dysregulation of cellular autophagy exacerbated podocyte injury in a mouse model of proteinuria. Our colleague reported that in puromycin-induced podocyte injury, the cellular autophagic process is disrupted, and p62 degradation is deficient. In contrast, treatment with an anti-ANGPTL3 antibody ameliorated the autophagic degradation deficiency by attenuating the impaired autophagosome-lysosome fusion, further reducing ROS production and attenuating podocyte apoptosis [9]. Dysfunctional autophagy has also been proposed by Ji *et al*. as a marker of podocyte apoptosis *in vitro* and *in vivo*. Antagonizing ANGPTL3 has been shown to almost completely protect a mouse model of NS by alleviating podocyte autophagy and inhibiting apoptosis [10]. Previous studies have reported that ANGPTL3 is involved in the functional and morphological regulation of mitochondria in the adipose tissue [11]. Therefore, anti-ANGPTL3-FLD monoclonal antibodies are expected to treat NS by affecting cellular autophagy and regulating mitochondrial function.

Interleukin-22 (IL-22), a member of the IL-10 cytokine family, functions as an anti-inflammatory and immunosuppressive factor. It binds to its receptor on the cell membrane to form a trimer and primarily activates the Janus Kinase / Signal Transducer and Activator of Transcription 3 (STAT3) signaling pathway, thereby mediating various biological functions such as suppressing inflammation and oxidative stress [12]. Additionally, IL-22 acts as a pro-regenerative cytokine that promotes the repair and regeneration of damaged cells and tissues. Treatment with recombinant IL-22 attenuates the severity of acute and chronic pancreatitis in mice by the induction of Bcl-2 and Bcl-X_L_, which bind to Beclin-1 and subsequently inhibit autophagosome formation [13]. In addition, IL-22 attenuates mitochondrial ROS accumulation and dysfunctional mitochondria by inducing the AMPK/AKT signaling pathway and PFBFK3 activity, ultimately improving renal function in mice with diabetic nephropathy [14]. Additionally, IL-22 may be involved in activating STAT3 and AKT phosphorylation in proximal tubular epithelial cells, to promote the upregulation of Bcl-2 and the downregulation of Bad levels, which ultimately ameliorated renal injury by resisting apoptotic processes in a mouse model of renal ischemia–reperfusion. Furthermore, accumulating data suggest that IL-22 has emerged as a fundamental mediator of epithelial homeostasis in the kidney by promoting tissue repair and regeneration while inhibiting oxidative stress.

Therefore, we hypothesized that the binding of anti-ANGPTL3 antibodies and IL-22 could regulate the autophagy process, attenuate mitochondrial damage, ameliorate oxidative stress injury, protect cells from apoptosis, repair damaged renal tissues, and delay renal disease progression. In previous studies, we have successfully constructed a novel anti-ANGPTL3/IL-22 fusion protein, which was shown to improve renal function in *db/db* mice by attenuating the inflammatory response and protecting podocytes. Accordingly, we applied the novel anti-ANGPTL3/IL-22 bifunctional protein in an adriamycin-induced NS mouse model to verify whether this protein could ameliorate ADRN.

## Materials and methods

### NS patient selection and non-NS subject recruitment

Human serum samples were collected from 24 patients with NS and 9 subjects without NS. The inclusion criteria for NS patients were as follows: meeting the diagnostic criteria for NS and having a glomerular filtration rate ≥90 ml/min. The exclusion criteria were severe infections, chronic kidney disease, tumors, and familial hypercholesterolemia. Non-NS subjects were recruited from among circumcised children with normal renal function and urine routine. The study was authorized by the Institutional Review Board of the Pediatric Hospital of Fudan University with an ethics code of (2021)-446, and all participants signed an informed consent form.

### Generation of the anti-ANGPTL3 antibody, mIL-22-Fc, and anti-ANGPTL3/IL-22 bifunctional fusion protein: production and functional validation

Following previously reported methods [15], the genes encoding the anti-ANGPTL3 antibody, mIL-22-Fc, and anti-ANGPTL3/IL-22 bifunctional fusion protein were successfully cloned and enzymatically digested with EcoRI and BamHI. They were then ligated into the pTT5 plasmid vector for amplification and transfected into CHO-S cells for eukaryotic expression. Finally, we collected the cell supernatants and purified the proteins using a Protein A affinity chromatography column. The humanized anti-ANGPTL3/IL-22 bifunctional fusion protein was produced using the same procedure.

Sodium Dodecyl Sulfate - Polyacrylamide Gel Electrophoresis (SDS-PAGE) was performed under reducing conditions using 10% Tris-glycine gels. Following electrophoresis, the proteins were visualized by staining with Coomassie brilliant blue (Invitrogen) and subsequently destained in a solution of 10% methanol and acetic acid.

Thermal stability measurements were performed on a NanoTemper PR.48 instrument over a temperature gradient of 20°C to 95°C. Following dilution of all samples in PBS buffer, the obtained data were fitted, with the Tm1 value defined as the melting point.

To assess the IL-22 activity of the anti-ANGPTL3/IL-22 bifunctional protein, mPTC cells were inoculated overnight and subsequently treated with 20 mM of the protein for durations of 2, 4, and 6 h. Following centrifugation, the supernatant from the cell lysates was collected for Western blot analysis.

### Animal model establishment

Male BALB/c mice (6–8 weeks old) were provided by the Changzhou Cavins Animal Laboratory Co. (Jiangsu, China) and housed under specific pathogen-free conditions. All the mice had access to reconstituted water and food.

For the fusion protein efficacy validation experiment, mice were randomized into six groups: the control (control), model (ADR), anti-ANGPTL3 antibody treatment (anti-ANGPTL3), IL-22 treatment group (mIL-22-Fc), anti-ANGPTL3/IL-22 bifunctional fusion protein treatment group (anti-ANGPTL3/IL-22), and anti-ANGPTL3 combined with mIL-22-Fc treatment (anti-ANGPTL3 + IL-22) groups. Saline was administered to the control group, and a single dose of adriamycin (10.5 mg/kg) was administered to the remaining mice via tail vein injection on Day 0. Mice in the treatment groups were intraperitoneally injected with anti-ANGPTL3 antibody (20 mg/kg), mIL-22-Fc (12 mg/kg), anti-ANGPTL3/IL-22 fusion protein (25.2 mg/kg), or a combination of anti-ANGPTL3 antibody (20 mg/kg) and mIL-22-Fc (12 mg/kg) twice weekly for 8 weeks. The humanized fusion protein was tested using the same model at the same dosage.

At the end of week 8, mouse serum was collected from the inner canthus for biochemical measurements. Mouse urine was collected from metabolic cages for biochemical measurements. Serum creatinine, urea nitrogen, total cholesterol (T-CHO), and serum albumin levels were measured using relevant test kits (Nanjing Jiancheng Company, China). Urinary microalbumin was detected using an ELISA kit (Nanjing Jiancheng Company), and the ratio of urine albumin-to-creatinine ratio (UACR) was calculated. For the subsequent histological examination, BALB/c mice (14–18 weeks old) were euthanized by 70% CO_2_ inhalation and then sacrificed by cervical dislocation.

All animal experiments were approved by the Ethics Committee of the School of Pharmacy at Fudan University. The experimental design and dosing regimens are presented in the schematic diagram.

### Renal histopathology

Kidneys were sectioned longitudinally with tissue shears, fixed adequately with 4% paraformaldehyde solution, embedded in paraffin, and cut into 4–5 μm thick sections. Hematoxylin and eosin (H&E), periodic acid Schiff (PAS), and Masson’s trichrome (Masson) staining were used to observe the morphological changes. The percentage of positively stained areas was then quantified using ImageJ software.

### Transmission electron microscopy analysis

The mice were sacrificed 8 weeks after drug administration. Briefly, kidney tissue was placed in 2.5% glutaraldehyde, washed with 0.01 M Phosphate Buffered Saline (PBS), and fixed with 1% osmium acid. Finally, the samples were dehydrated in a gradient ethanol series and acetone and embedded with Epon812. Four glomeruli were randomly selected per mouse, and two electron micrographs were taken from each mouse. Transmission electron microscopy (TEM) images of podocytes, mitochondria, and autolysosomes in the kidney tissue were analyzed using ImageJ software.

### Immunofluorescence analysis

Mouse kidney tissues were collected and subjected to immunofluorescence staining for the active form of integrin β3 (Kerafast, USA, Dilution ratio 1:200), nephrin (Servicebio, China, 1:200), fibronectin (Servicebio, Wuhan, China, 1:100), α-SMA (Servicebio, 1:300), synaptopodin (Servicebio, 1:300), TOM20 (Servicebio, 1:150), and cleaved caspase 3 (CST, USA, 1:400) to observe the expression of corresponding proteins reflecting podocyte injury, fibrosis, mitochondrial damage, and apoptosis. Cell nuclei were labeled with Hoechst 33342. The dilutions were purchased from Servicebio (Wuhan, China). Positively stained areas were counted using ImageJ software, and the percentages of positively stained areas in the images were calculated.

### Immunohistochemical analysis

Immunohistochemical staining for ANGPTL3 (Abcam, Lon., UK, 1:200) was performed to explore the correlation between ANGPTL3 expression and NS progression. Moreover, immunohistochemical staining of podocin (Servicebio, 1:200), KIM-1 (Sino Biological, 1:800), Alpha-smooth muscle actin (α-SMA) (Servicebio, 1:200), Bcl2 (Servicebio, 1:500), and LC3II (Servicebio, 1:100) was performed to assess the role of anti-ANGPTL3/IL-22 fusion proteins in improving podocyte and tubular function, as well as alleviating fibrosis, apoptosis, and regulating autophagy. Nuclei were labeled with Hoechst 33342. Positively stained areas were evaluated using ImageJ software, and the percentage of positively stained areas in the entire image was calculated.

### Western blot analysis

Total proteins were collected after the homogenization of kidney tissues. Equal amounts of proteins (20 μg per well) were subjected to SDS-PAGE and incubated with antibodies specific for p-Stat3 (CST, 1:1000), LC3II (1:1000) and GAPDH (CST, 1:1000). The target proteins were visualized using enhanced chemiluminescence substrates. ImageJ software was used to quantify the resulting gray band values.

### ELISA

Serum superoxide dismutase (SOD) activity and malondialdehyde (MDA) levels were measured using SOD and MDA assay kits (Nanjing Jiancheng), respectively, according to the manufacturer’s instructions. The optical density values of SOD and MDA were measured at 460 and 532 nm, respectively, using an enzyme-labeling instrument (Tecan Infinite F50, Switzerland).

We then used Enzyme-Linked Immunosorbent Assay (ELISA) to test the binding site of the humanized anti-ANGPTL3/IL-22 fusion protein on the N-terminal CCD or C-terminal FLD. Briefly, each well was coated with 100 ng of hANGPTL3-CCD-His or hANGPTL3-FLD-His on an ELISA plate at 4°C for 12 h. After washing with Phosphate Buffered Saline with Tween 20 (PBST) and blocking with 3% bovine serum albumin at 37°C for 1 h, the humanized anti-ANGPTL3/IL-22 fusion protein, diluted in a three-fold gradient starting from a concentration of 100 nM, was added to the plate and incubated at 37°C for 1 h. After washing with PBST, anti-human IgG1-Fc antibody with Horseradish Peroxidase (HRP) (Thermo, 1:10 000) was added to the plate to react with TMB (Beyotime, China). The absorbance of the wells at 450 nm (OD_450_) was then determined.

### mRNA sequencing and bioinformatics analysis

Total RNA was extracted from renal tissues using the RNAmini kit (Qiagen, Germany). RNA quality was assessed by gel electrophoresis and with Qubit fluorometry (Thermo, Waltham, MA, USA). Each group contained three biological replicated samples that were separated into independent pool. Strand-specific libraries were constructed using the Stranded mRNA-seq(NR602)(Vazyme, Nanjing, China), and sequencing was carried out by 2 × 150 bp paired-end using the lllumina NovaSeq 6000 instrument. Double-stranded cDNA was then purified with AMPure XP beads and then modified with End Repair Mix to add poly-A tails and sequencing connectors. Subsequently, the cDNA was converted to single-stranded DNA using the USER enzyme, and Polymerase Chain Reaction (PCR) amplification was performed for 15 cycles to form a cDNA library. Finally, PCR clustering was performed on a cBot instrument, and sequencing was completed in the 2 × 151 mode.

Next, we used the DESeq2 (1.30.1) package to normalize the count value of the expression matrix and perform differential expression analysis among the different groups. Based on the results of the normalized expression matrix and differential gene analysis, we then used the ggplot2 (3.3.6) package to draw principal component analysis (PCA) plots and scatter plots, respectively. Heatmaps of differential genes related to oxidative stress, mitochondrial function, autophagy, and apoptotic pathways were plotted using the Complex Heatmap (2.20.0) package. Next, Kyoto Encyclopedia of Genes and Genomes (KEGG) pathway analysis was performed based on the results of the differential gene analysis using ClusterProfiler (4.13.4) and org.Mm.eg.db (3.19.1) packages. All the above codes are based on R (4.3).

### Humanization of the anti-ANGPTL3/IL-22 fusion protein

Based on the original murine antibody sequence, homology modeling was performed to analyze the framework amino acids within a 5-Å range of the complementary determining regions (CDRs), which typically influence the conformation or antigen-binding activity of the CDRs. Human germline sequences were obtained via international ImMunoGeneTics information system (IMGT) analysis, from which the human germline framework was selected and spliced using the original CDRs, leading to an initial version of the humanized antibody. By comparing the sequences and homology models of the antibody before and after design, the amino acids were adjusted and replaced based on minimizing heterogeneity while maintaining primary antibody activity. The final sequence of the humanized antibody was determined after repeated modifications and optimizations. Subsequently, human IL-22 was fused to the C-terminus of the heavy chain of the anti-ANGPTL3 hAb via a linker sequence (Gly4Ser)_3_ to obtain a humanized anti-ANGPTL3/IL-22 fusion protein.

### Surface plasmon resonance

Surface plasmon resonance (SPR) was used to determine the affinity of the humanized anti-ANGPTL3/IL-22 fusion protein for human-derived or mouse-derived ANGPTL3 using a Biacore T200 system (GE Healthcare, USA). First, we immobilized ANGPTL3 on a CM5 chip (GE Healthcare) at ~60 RU by regulating the capture time. Next, the humanized anti-ANGPTL3/IL-22 fusion protein was diluted with Ethylenediaminetetraacetic acid and Surfactant P20 buffer and passed through the chip for binding at 120 s and dissociation at 480 s. After each round, glycine-HCl buffer (pH 2.5) was used for regeneration. The affinity constant (KD) was determined by calculating the ratio of the dissociation constant (Kd) to the binding constant (Ka).

### Dynamic light scattering analysis

Dynamic light scattering was performed to determine the size and size distribution of the target protein in the solution by measuring rapid changes in the intensity of the laser light scattered by molecules or particles. Uncle (Unchained Labs, USA), an all-in-one biological stability screening platform, was used to evaluate protein diameter, polydispersity, thermal stability, and colloidal stability by using laser-excited light at 266 and 473 nm. For the melting temperature (Tm) and the aggregation temperature (Tagg), protein samples were heated from 15°C to 95°C using a 0.3°C/min ramp rate, and data were collected every 5 s. The raw data obtained were fitted to a sigmoidal shape via Uncle Analysis.

### Real-time PCR and ATP analysis

Real-time PCR (R&D Systems, USA) was used to detect changes in Nephrin and KIM-1 mRNA expression in renal tissues of mice from each group. Total RNA (2.0 μg) was denatured at 65°C for 5 min and reverse transcribed into cDNA at 42°C for 30 min using reverse transcriptase. Then, 5 μl of cDNA product was taken and added to a mixture containing 10× PCR buffer (50 pmol each of upstream and downstream primers), 0.2 mmol/L dNTP, 2.0 mmol/L MgCl₂, and 1 U *Taq* DNA polymerase. PCR amplification was performed under the following conditions: pre-denaturation at 94°C for 2 min, followed by 30 cycles of denaturation at 94°C for 30 s, annealing at 58.8°C for 30 s, extension at 72°C for 40 s, and a final extension at 72°C for 5 min. Primer sequences were as follows: Nephrin forward 5′-TACCACCAGCAT TCCACG-3′, reverse 5′-GGGCTCGGCTGTATGTATT-3′, KIM-1: forward 5′-TCACCTCAG CCAGCAGAAACCC-3′, reverse 5′-CACCCAAAAGAGCAAGAAGCAC-3′, GAPDH: forward 5′-CTCTACCCCACGGCAAAGTTCAA-3′, reverse 5′-GGATGACCTTGCCCACA GC-3′. PCR products of Nephrin and KIM-1 (10 μl each) were subjected to 1.5% agarose gel electrophoresis. The gel was imaged, and the optical density was measured. mRNA expression levels were semi-quantitatively analyzed relative to GAPDH.

The ATP assay was performed according to the manufacturer’s protocol. Briefly, the luciferase-luciferin working solution was prepared and equilibrated to room temperature. A standard curve was generated using serial dilutions of ATP (0–100 μM). Cell or tissue samples were lysed, centrifuged, and the supernatant was collected. An equal volume of the working solution was added to each sample or standard in a microplate. After a brief dark incubation, luminescence was measured using a microplate reader. ATP concentrations were calculated based on the standard curve.

### Statistical analysis

All the data are expressed as the means ± standard errors of the mean. Differences between groups were assessed using Student’s *t-*test or one-way Analysis of Variance (ANOVA). Pearson’s correlation analysis was performed using SPSS Statistics for Windows version 20. Statistically significant differences are indicated as ^*^P < .05, ^**^P < .01, and ^***^P < .001.

## Results

### ANGPTL3 upregulation and IL-22 downregulation are observed in patients with NS and NS mice

**Figure 1:**
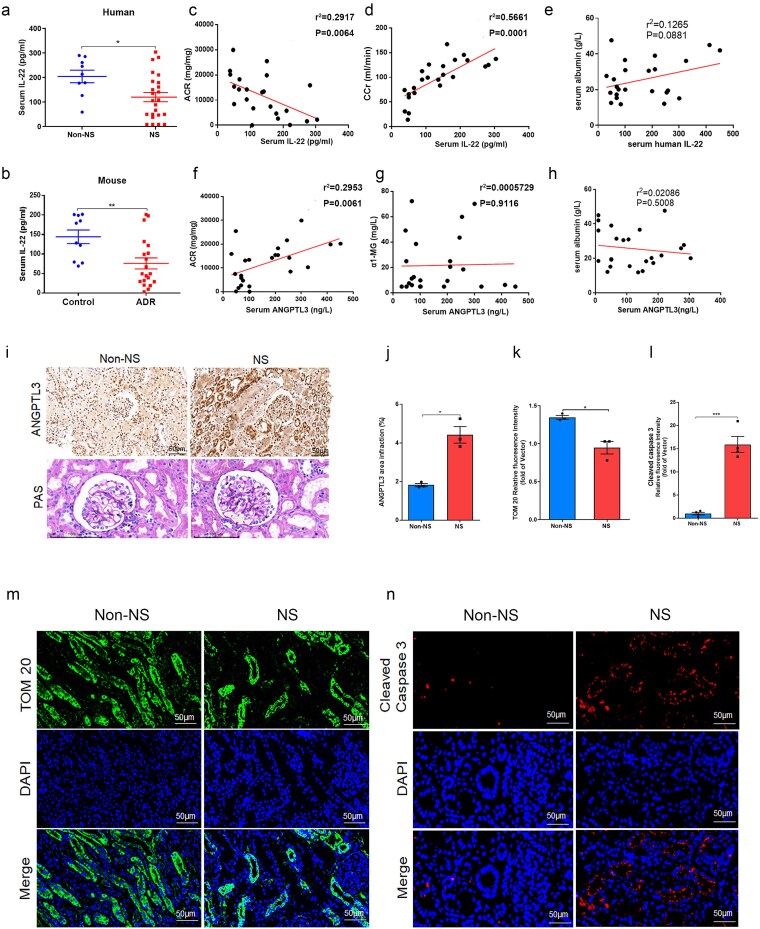
The expression of ANGPTL3 and IL-22 in NS; (a) serum levels of IL-22 from healthy subjects (*n* = 9) and patients with NS (*n* = 24); (b) serum levels of IL-22 in healthy mice (*n* = 10) and ADR mice (*n* = 20); (c) Pearson correlation between serum human IL-22 and the ACR; (d) Pearson correlation between serum IL-22 and Ccr; (e) Pearson correlation between serum IL-22 and serum albumin; (f) Pearson correlation between serum ANGPTL3 and ACR; (g) Pearson correlation between serum ANGPTL3 and α1-MG; (h) Pearson correlation between ANGPTL3 and serum albumin; (i) representative PAS images of kidney samples and immunohistochemical staining of ANGPTL3 in kidney samples; scale bars: PAS, 100 μm; ANGPTL3, 50 μm; (j) quantitative analysis of ANGPTL3 expression; (m) immunofluorescence analysis of TOM20 expression; scale bar: 50 μm; (n) immunofluorescence analysis of cleaved caspase 3 expression; scale bar: 50 μm; (l, k) statistical assessment of the levels of TOM20 and cleaved caspase 3; ^*^P < .05, ^**^P < .01, ^***^P < .001 ^****^P < .0001.

Our data revealed that serum IL-22 levels were significantly lower in patients with NS and NS mice than in the healthy controls (Fig. 1a and b). In addition, we found that serum IL-22 levels were negatively correlated with the UACR and positively correlated with creatinine clearance (Ccr) and serum albumin level (Fig. 1c–e).

Previous studies have shown that ANGPLT3 expression is elevated in the serum of patients with NS [15]. Our data verified increased ANGPTL3 expression in the kidneys of patients with NS, whereas expression was low in normal renal tissue. ANGPTL3 was predominantly localized to podocytes, with mild expression also observed in renal tubules (Fig. 1i and j). We further investigated the associations between ANGPTL3 levels and the UACR, serum albumin level, and urinary α1-microglobulin level. The results revealed that serum ANGPTL3 was positively correlated with the UACR and negatively correlated with serum albumin levels but not with urinary α1-microglobulin (Fig. 1f–h).

Taken together, our results suggest that the upregulation of ANGPTL3 and downregulation of IL-22 may be associated with NS progression. Therefore, we hypothesized that the co-administration of an ANGPTL3 antibody and IL-22 could ameliorate NS. In addition, we found that the expression levels of apoptosis-associated protein cleaved caspase-3 were significantly elevated, whereas the expression of the mitochondrial membrane protein TOM20 was reduced in the kidney tissues of patients with NS (Fig. 1k–n), suggesting that mitochondria were damaged, and apoptosis was exacerbated to a certain extent in patients with NS.

### The bioactivity of anti-ANGPTL3/IL-22 and its protective effect in NS mice

**Figure 2:**
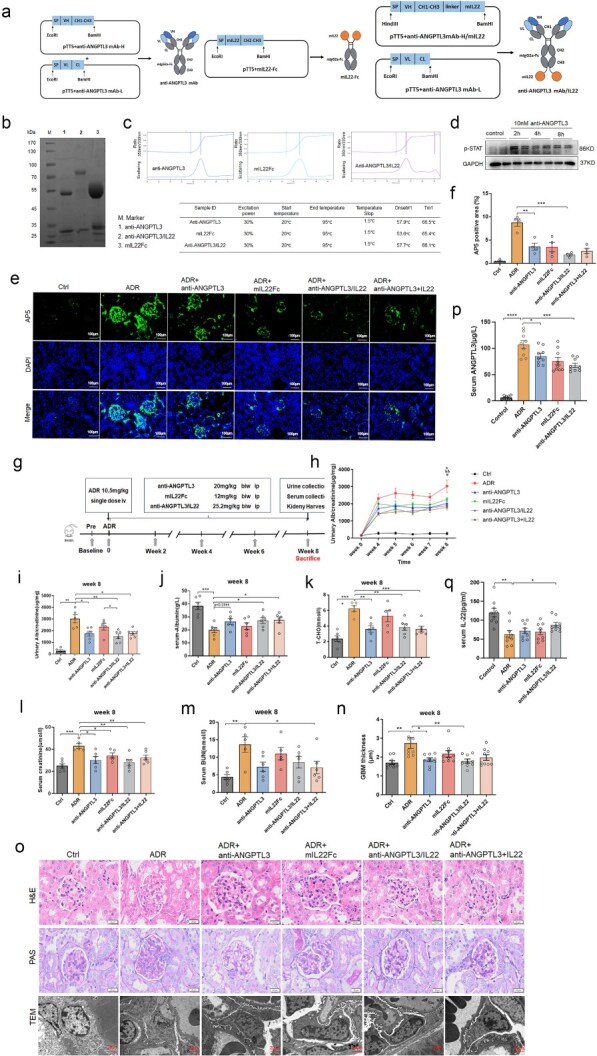
Anti-ANGPTL3/IL-22 fusion protein reduces renal injury and improves kidney function in ADR mice; (a) transfection and purification of anti-ANGPTL3, mIL-22-Fc, and anti-ANGPTL3/IL-22 bifunctional fusion proteins using genetic engineering techniques; (b) SDS-PAGE analysis of anti-ANGPTL3 (lane 1), anti-ANGPTL3/IL-22 (lane 2), and mIL-22-Fc (lane 3) under reducing condition (anti-ANGPTL3: ~150 KD, IL-22Fc: ~90 KD, anti-ANGPTL3/IL-22: ~190 KD); (c) thermal stability analysis of anti-ANGPTL3, anti-ANGPTL3/IL-22, and mIL-22-Fc; (d) after mouse proximal tubular epithelial cells (mPTC) were incubated overnight, 10 nM anti-ANGPTL3/IL-22-induced phosphorylation of STAT3 (p-STAT3) was analyzed by western blot at different times; densitometric values of p-STAT3 expression after 2 h, 4 h, and 6 h (*n* = 4); (e) mice were first treated with anti-ANGPTL3, mIL-22-Fc, anti-ANGPTL3/IL-22. Then, the kidneys were stained with AP5 and Hoechst 33342 to assess intracellular exposed PSI epitopes (scale bar: 100 μm); (f) statistical analysis of AP5 levels by immunofluorescence staining (*n* = 4); (g) animal experiment flowchart; (h) ACR levels over 8 weeks; (i) ACR levels on week 8; (j) serum albumin levels; (k) serum T-CHO levels; (l) serum creatinine levels; (m) serum BUN levels; (n) TEM quantification of GBM thickness; (o) Representative H&E (top) and PAS (middle) staining images of kidney samples from ADR mice; scale bar: 20 μm; bottom: representative electron microscopy images of ADR mouse glomeruli; (p) and (q) changes in serum ANGPTL3 and IL-22 expression levels after treatment with anti-ANGPTL3, mIL-22-Fc, and anti-ANGPTL3/IL-22, respectively; scale bar: 20 μm. ^*^P < .05, ^**^P < .01, ^***^P < .001, ^****^P < .0001.

Genetic recombination technology was used to produce bifunctional fusion proteins. SDS-PAGE under reducing conditions results showed the expected molecular weight bands and high purity of anti-ANGPTL3, mIL-22-Fc, and anti-ANGPTL3/IL-22 fusion protein (Fig. 2a and b). The Tm1 values of anti-ANGPTL3 and anti-ANGPTL3/IL-22 fusion protein were 66.5°C and 66.1°C, respectively, indicating that the fusion protein had the same favorable thermal stability as the monoclonal antibody (Fig. 2c). To validate the biological activity of anti-ANGPTL3/IL-22, it inhibits the exposure of the Plexin-Semaphorin-Integrin (PSI) epitope by blocking the binding between integrin αvβ3 and ANGPTL3, and under adriamycin treatment, it exerts the same effect on mouse kidneys as anti-ANGPTL3 (Fig. 2e and f). Besides, immunoblots analysis confirmed that both anti-ANGPTL3/IL-22 and mIL-22-Fc could activate STAT3 phosphorylation in mPTC in a time-dependent manner (Fig. 2d). Together, the anti-ANGPTL3/IL-22 was successfully constructed and maintained the dual biological effect of anti-ANGPTL3 and IL-22.

A single dose of adriamycin administered via tail vein injection was used to establish an experimental model of NS; the subsequent administration regimen is shown in Fig. 2g. While both anti-ANGPTL3 and mIL-22-Fc therapy decreased ACR levels, the fusion protein offered only a slight edge, with no markedly greater benefit observed over the individual treatments (Fig. 2h and i). In addition to changes in ACR levels, fusion protein administration significantly decreased serum creatinine, blood urea nitrogen, and T-CHO levels but increased serum albumin levels (Fig. 2j–m). More importantly, these results indicate that anti-ANGPTL3/IL-22 fusion protein treatment offered greater advantages in improving renal function and attenuating renal injury than anti-ANGPTL3 monoclonal antibody or mIL-22-Fc treatment alone, highlighting its prominent protective effect on renal function.

In addition, after 8 weeks of anti-ANGPTL3/IL-22 fusion protein treatment, H&E and PAS staining revealed that the glomerular vascular alignment of the fusion protein-treated group of mice was more evident than that of the model group, the degree of glomerular adhesion was reduced, and the effect was greater than that of the anti-ANGPTL3 antibody and mIL-22-Fc treatment (Fig. 2o). Consistent with the histological findings, TEM analysis indicated that treatment with the bifunctional protein led to a modest reduction in glomerular basement membrane (GBM) thickness compared to the model group, whereas the anti-ANGPTL3 or mIL-22-Fc alone showed only minimal improvement (Fig. 2n and o). These data suggest that the anti-ANGPTL3/IL-22 fusion protein significantly improved renal function and reduced renal injury in an ADR-induced mouse model of NS. Finally, changes in the serum levels of ANGPTL3 and IL-22 were measured in each group of mice (Fig. 2p and q). The results indicated that treatment with the fusion protein played an important role in both elevating IL-22 and reducing ANGPTL3 levels, thereby laying the foundation for its protective effects in the kidney.

### Anti-ANGPTL3/IL-22 bifunctional fusion protein attenuates glomerular and tubular injury and ameliorates renal fibrosis in NS mice

**Figure 3:**
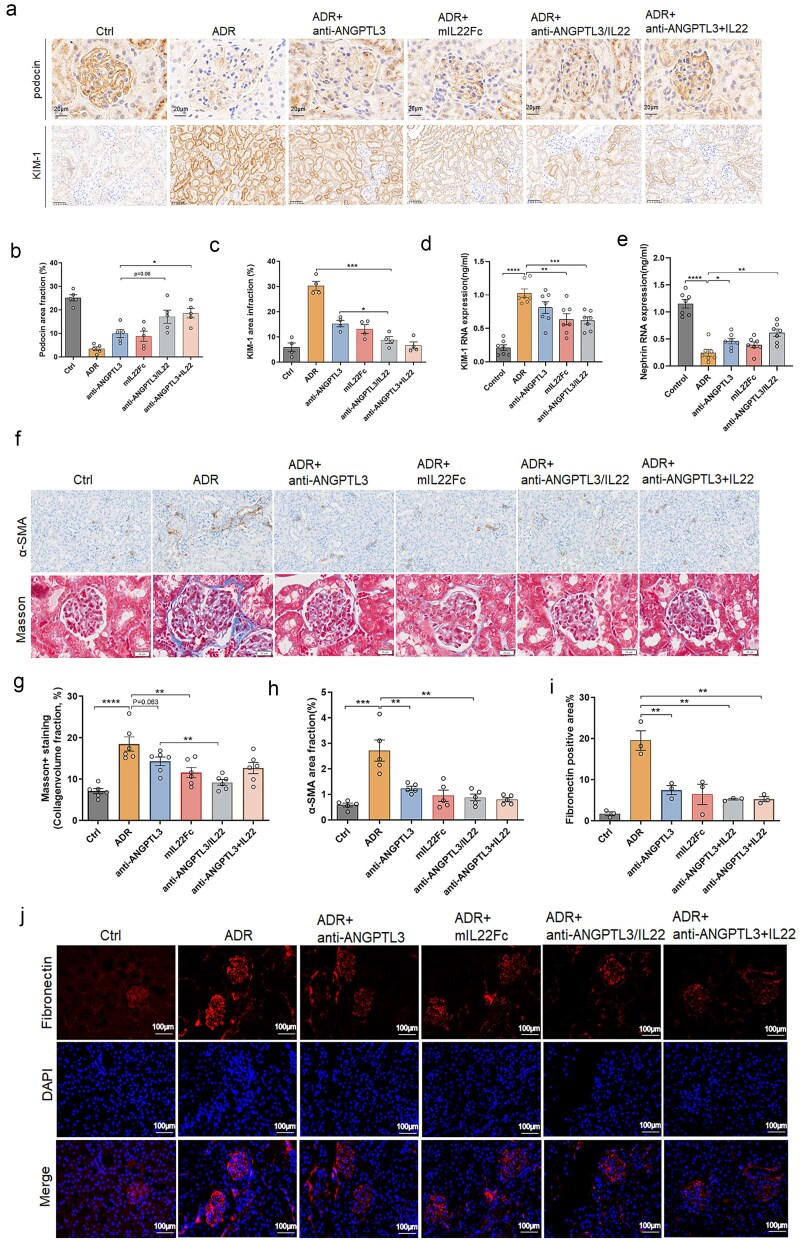
The effects of anti-ANGPTL3/IL-22 bifunctional fusion protein in NS mice; (a) immunohistochemical images of podocin and KIM-1 in kidney sections; scale bars: 20 μm and 100 μm; (b, c) quantitative analysis of podocin and KIM-1 expression; (d) statistical analysis of KIM-1 expression by RT-PCR. (e) statistical analysis of Nephrin expression by RT-PCR; (f) top: immunohistochemical images of α-SMA expression; scale bar: 100 μm; bottom: representative Masson staining images of mouse kidney samples; scale bar: 20 μm; (g) quantification of Masson staining in kidney sections; (h) quantitative analysis of α-SMA expression; (i) quantification of fibronectin expression in kidney sections; (j) immunofluorescence images of fibronectin expression in kidney sections; scale bar: 100 μm. ^*^P < .05, ^**^P < .01, ^***^P < .001, ^****^P < .0001.

The activation of integrin αvβ3 in glomerular podocytes has been shown to cause podocyte damage and proteinuria after adriamycin treatment [16]. The expressions of podocin, a glomerular slit diaphragm marker protein, and KIM-1, a tubular epithelial cell injury biomarker, were also assessed to verify their effects on glomerular and tubular functions. The proportion of positive areas in the kidney sections revealed that the levels of podocin were elevated, whereas the levels of KIM-1 were lower in the anti-ANGPTL3 and mIL-22-Fc intervention group than in the ADR group, suggesting that the injury to the glomerular slit diaphragm and tubular epithelial cells was repaired (Fig. 3a–c). To investigate the effects of anti-ANGPTL3/IL-22 on glomerular and tubular functions, real-time PCR was used to detect the expression of Nephrin and KIM-1 proteins. The results showed that treatment with anti-ANGPTL3 decreased the expression of KIM-1 and upregulated the expression of nephrin, which are significant improvements in both markers. In terms of promoting the recovery of tubular function and glomerular functional proteins, the combined treatment demonstrated superior efficacy, while the application of the monoclonal antibody or mIL-22 alone resulted in only moderate repair (Fig. 3d and e).

The effect of anti-ANGPTL3/IL-22 on renal fibrosis in ADR group was further explored. Excessive extracellular matrix deposition contributes to renal fibrosis, eventually resulting in renal failure in patients with NS. Therefore, we investigated whether the anti-ANGPTL3/IL-22 fusion protein could inhibit renal fibrosis. Our results revealed that renal collagen accumulation was reduced after 8 weeks of treatment with the anti-ANGPTL3 antibody, mIL-22-Fc, and the anti-ANGPTL3/IL-22 fusion protein, as shown by Masson staining, compared to that in the ADR group (Fig. 3f–h). Moreover, our findings further indicated that the administration of the anti-ANGPTL3/IL-22, anti-ANGPTL3 + IL-22, and mIL-22-Fc treatments all mildly reversed the high expression of the fibrosis-related proteins α-SMA and fibronectin in NS renal tissues. Anti-ANGPTL3 treatment reduced the levels of these proteins but not as much as in the fusion protein group (Fig. 3j–i). Taken together, these results indicated that proteinuria and renal fibrosis improved to some extent after anti-ANGPTL3/IL-22 fusion protein treatment, demonstrating the combined protective effect of anti-ANGPTL3 and mIL-22-Fc on the kidney.

### Anti-ANGPTL3/IL-22 bifunctional fusion protein maintains mitochondrial functional homeostasis and attenuates oxidative stress

**Figure 4:**
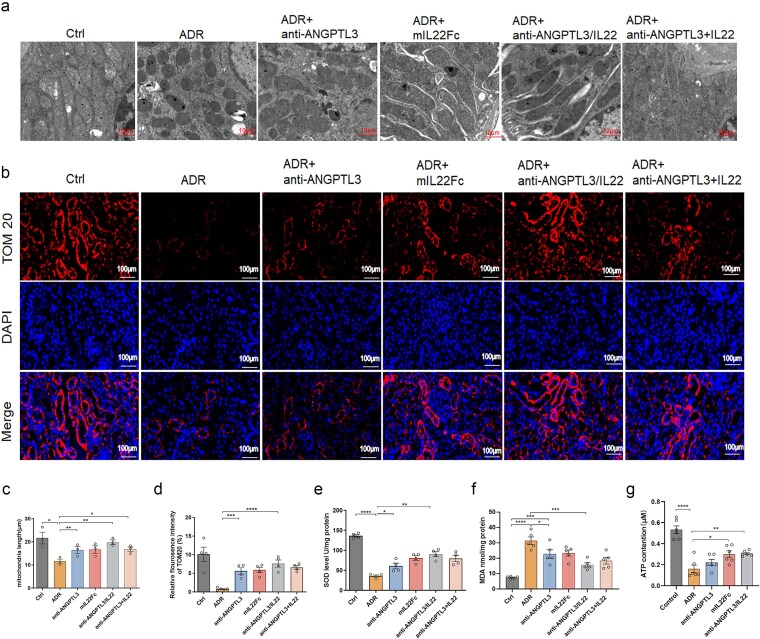
Anti-ANGPTL3/IL-22 fusion protein maintains mitochondrial functional homeostasis. (a, c) representative electron microscopy images of mitochondria in the glomerulus of ADR mice and statistical analysis of mitochondrial length; scale bar: 10 μm; mitochondria swelling, vacuolization, and cristae fragmentation in podocytes were observed in ADR nephropathy mice; (b) immunofluorescence images of TOM20 expression in kidney sections; scale bar: 100 μm; (d) quantitative analysis of TOM20 expression; (e, f) quantitative analysis of expression of SOD and MDA in kidney sections; (g) quantitative analysis of expression of ATP in kidney sections; ^*^P < .05, ^**^P < .01, ^***^P < .001, ^****^P < .0001.

Previous studies have shown that ANGPTL3 induces oxidative stress and IL-22 inhibits oxidative stress; however, the role of ANGPTL3 in oxidative stress in the kidney has not been investigated. The role of the mitochondria is central to oxidative stress. First, we used TEM to detect the morphological and structural changes in podocyte mitochondria, which were all diminished after ADR treatment, exhibiting broken internal cristae and vacuolar degeneration. Mitochondrial morphology and arrangement were significantly improved following anti-ANGPTL3/IL-22 treatment, surpassing the improvements observed in the anti-ANGPTL3 and mIL-22-Fc combination groups (Fig. 4a and c). Additionally, we examined the expression of TOM20, a key receptor subunit of the Translocase of the outer mitochondrial membrane (TOM) complex that mediates the initial step of mitochondrial protein transport and plays a critical role in apoptosis, pyroptosis, and autophagy *in vivo*. After 8 weeks of anti-ANGPTL3/IL-22 treatment, TOM20 expression significantly improved, mitochondrial function was repaired, renal function was promoted, and the overall therapeutic effect was greater than that in the anti-ANGPTL3 and mIL-22-Fc monotherapy groups (Fig. 4b and d). Further, we evaluated the changes in the levels of oxidative stress markers, such as MDA and SOD. Our results showed that the anti-ANGPTL3/IL-22, anti-ANGPTL3, and mIL-22-Fc groups all exhibited significantly increased levels of SOD and reduced levels of MDA in the kidneys, with the bifunctional protein group showing the slightly pronounced effects (Fig. 4e and f). To evaluate mitochondrial function, we measured ATP levels in the kidneys of mice from each group. The results showed that the bifunctional fusion protein treatment group exhibited a moderate increase in tissue ATP content and promoted the recovery of mitochondrial function, whereas the groups treated with anti-ANGPTL3 or IL-22 alone showed slightly reduced effects. These findings are consistent with the results obtained from SOD and MDA assays (Fig. 4g). These data suggest that anti-AGPTL3/IL-22 bifunctional fusion proteins are important for maintaining mitochondrial function to alleviate oxidative stress.

### Anti-ANGPTL3/IL-22 bifunctional fusion protein inhibits apoptosis and alleviates autophagy

**Figure 5:**
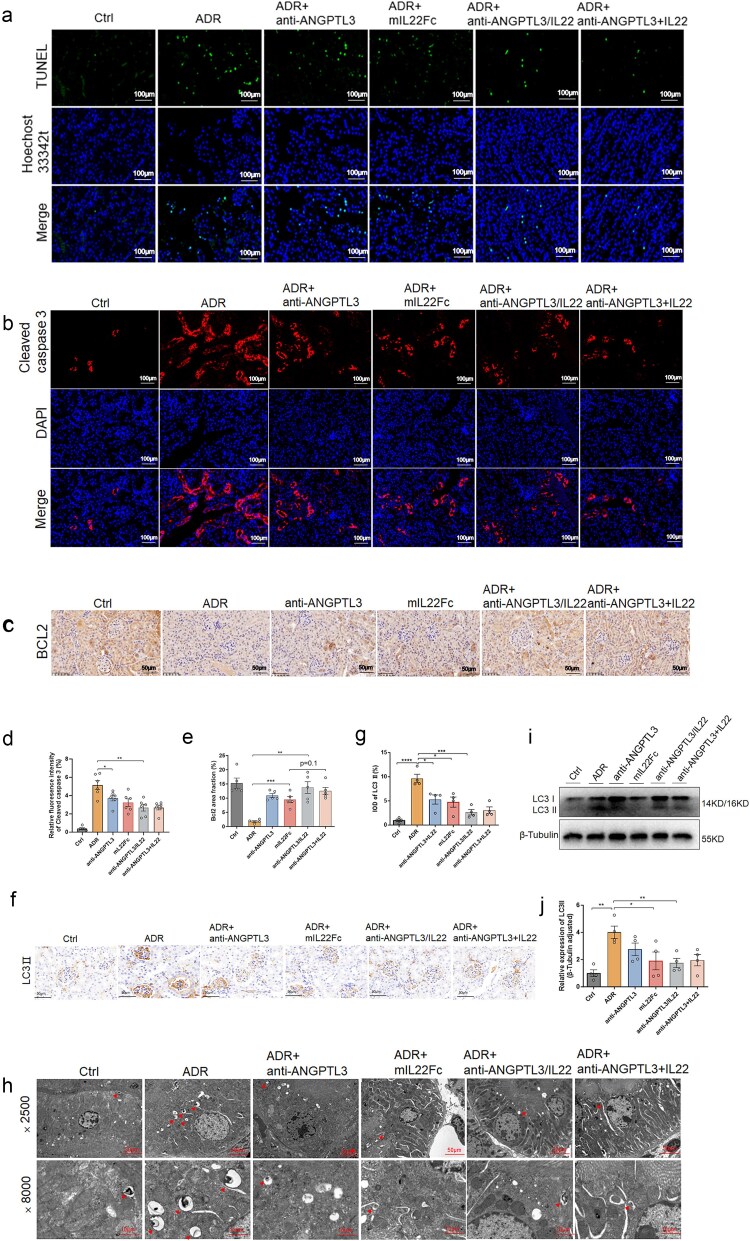
Anti-ANGPTL3/IL-22 treatment attenuates renal cell apoptosis and maintains intracellular environmental homeostasis; (a) representative TUNEL staining images of kidney tissues in each treatment group; (b) immunofluorescence images of cleaved caspase 3 expression in kidney sections; scale bar: 100 μm; (c) immunohistochemical images of Bcl2 expression; scale bar: 100 μm; (d) quantitative analysis of cleaved caspase 3 expression in the immunofluorescence staining images; (e) quantitative analysis of Bcl2 expression in the immunohistochemical staining images; (f) immunohistochemical images of LC3II; scale bar: 100 μm; (g) quantitative analysis of LC3II expression; (h) representative electron microscopy images of newly formed autophagy-fused lysosomes in podocytes in the different treatment groups; (i) detection of LC3II expression in kidney tissue by western blot; (j) quantitative statistical analysis of LC3II expression levels in renal tissue; ^*^P < .05, ^**^P < .01, ^***^P < .001, ^****^P < .0001.

To assess the level of apoptosis in renal cells of ADR group, we performed Terminal deoxynucleotidyl transferase dUTP nick-end labeling (TUNEL) staining and verified the expression of apoptosis-related proteins in the kidneys. TUNEL staining of kidney tissues showed that the application of anti-ANGPTL3, mIL-22-Fc, and anti-ANGPTL3/IL-22 treatments for nephropathy reduced the number of apoptotic cells in the kidneys, which was especially obvious after anti-ANGPTL3/IL-22 treatment and their combination (Fig. 5a). We then measured the expression of the pro-apoptotic protein cleaved caspase-3 and the anti-apoptotic protein Bcl-2, which regulate the mitochondrial apoptotic pathway. In the kidneys of ADR group, cleaved caspase-3 levels were significantly increased and Bcl-2 levels were decreased, which were partially reversed by anti-ANGPTL3/IL-22 fusion protein treatment. These results show that mIL-22-Fc provides superior efficacy in reducing Caspase-3 levels compared to anti-ANGPTL3, but was slightly less effective in increasing Bcl-2 expression, highlighting the need for a combined approach (Fig. 5b–e).

To observe the changes of autophagy after adriamycin-induced renal tissue injury, we detected the expression of LC3-II to reflect the status of autophagy. Immunohistochemical results revealed that the expression of LC3-II was significantly higher in the renal tissues of the ADR group than in those of the control group (Fig. 5f and g). We also detected LC3II expression in the kidney tissue by western blot (Fig. 5i and j). Intervention with anti-ANGPTL3, mIL-22, and anti-ANGPTL3/IL-22 proteins reduced the accumulation of LC3-II in renal tissues to varying extents, among which the fusion protein or combination treatment had the greatest effect. This result suggests that anti-ANGPTL3/IL-22 fusion proteins can relieve ADR-induced LC3-II accumulation, although it remains unclear whether the accumulation is caused by autophagy activation or a block in the completion of the autophagy pathway.

We combined the results of TEM to assess autophagy status. Compared to the control group, the number and volume of intracellular autolysosomes increased after ADR-induced podocyte injury. In contrast, the number and volume of autolysosomes with increased intracellular volume were reduced after intervention with the anti-ANGPTL3 antibody, mIL-22, and anti-ANGPTL3/IL-22. The effect in the bifunctional protein intervention group was more pronounced than in the other groups (Fig. 5h). These results infer that the intervention of ADR activates autophagy which is alleviated by the treatment of fusion proteins.

### Transcriptomic analysis reveals the potential molecular basis for the greater efficacy of the anti-ANGPTL3/IL-22 bifunctional fusion protein

**Figure 6:**
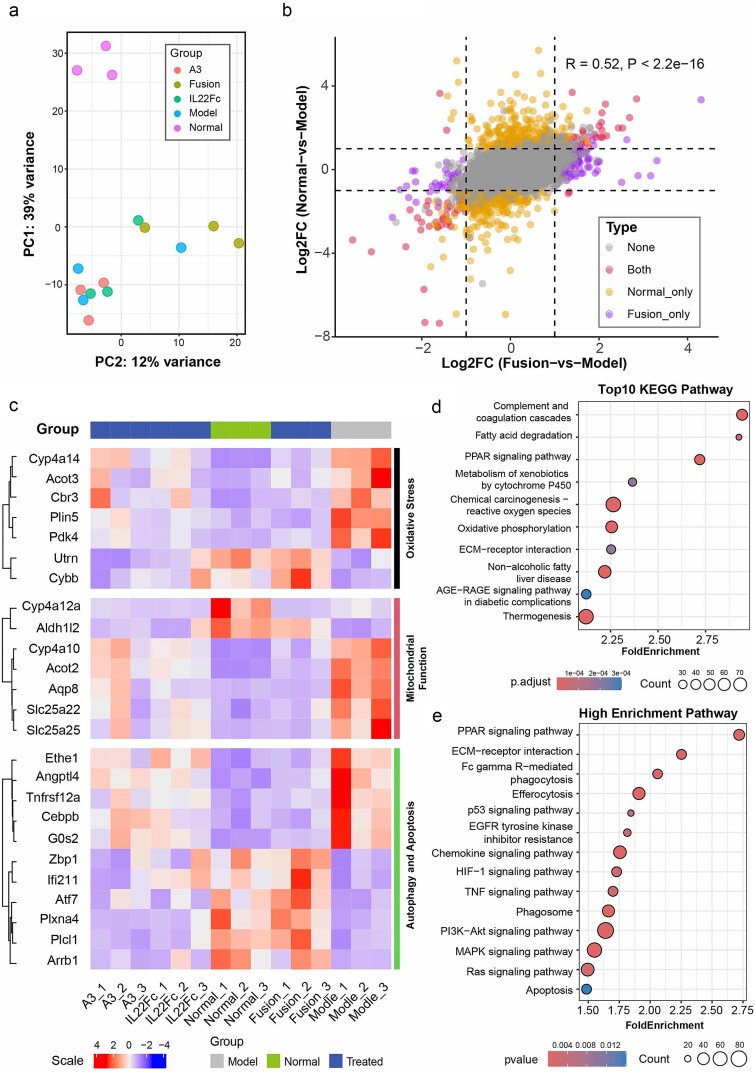
Transcriptomic analysis of ADR mice treated with anti-ANGPTL3/IL-22; (a) principal component analysis plot showing the distribution of samples from different subgroups after dimensionality reduction; (b) differentially expressed genes (DEGs) between anti-ANGPTL3/IL-22 and ADR groups and DEGs between the Control and ADR groups were projected on the x-axis and y-axis, respectively, according to their log2FC values. The correlation coefficients and p-values of the DEGs between two groups are indicated; (c) expression spectra of oxidative stress-, mitochondrial function-, autophagy-, and apoptosis-related gene signatures are visualized as a heatmap; (d) Top 10 and (e) highly enriched KEGG signaling pathways of the DEGs in the anti-ANGPTL3/IL-22 group compared to the ADR group.

To elucidate the molecular changes before and after modeling and anti-ANGPTL3/IL-22 treatment, we analyzed mouse kidney samples via transcriptome sequencing. The PCA plot demonstrated distinct clustering of samples from different subgroups after downscaling, which showed that the ADR and Control groups could be significantly differentiated and that the anti-ANGPTL3/IL-22 group had better therapeutic outcomes than the anti-ANGPTL3 monoclonal antibody and mIL-22-Fc monotherapy groups (Fig. 6a). Differentially expressed genes (DEGs) between the anti-ANGPTL3/IL-22 and ADR groups and between the Control and ADR groups were projected onto the x- and y-axes, respectively, according to their log2FC values. The correlations and p-values of DEGs between the two groups were annotated (Fig. 6b). Compared to the other two treatment groups, the anti-ANGPTL3/IL-22 group showed a stronger correlation with the control DEGs (Supplementary Figs S1and S2). A heat map of DEGs related to oxidative stress, mitochondrial function, autophagy, and apoptosis revealed significant differences between the ADR and Control groups. These differences were partially reversed by anti-ANGPTL3 or mIL-22-Fc treatment and significantly reversed upon treatment with the anti-ANGPTL3/IL-22 protein, with gene expression profiles closely approximating those of the Control group. These results suggest a molecular basis for the superior efficacy of the fusion protein compared to that of monotherapy (Fig. 6c).

Compared to the ADR group, the top 10 KEGG signaling pathways of the DEGs in the anti-ANGPTL3/IL-22 group were identified, including pathways related to autophagy and oxidative stress. These results demonstrate changes in the major pathways before and after treatment, including autophagy-related, apoptosis-related, and oxidative stress-related pathways (Fig. 6d and e). Collectively, our transcriptomic analysis further revealed the potential molecular basis for the greater efficacy of the anti-ANGPTL3/IL-22 bifunctional fusion protein than that of the monotherapy. Meanwhile, the therapeutic effect of this protein on NS could be associated with the regulation of autophagy, mitochondrial function, and apoptosis signaling pathways.

### The renoprotective efficacy of humanized anti-ANGPTL3/IL-22 bifunctional fusion protein in ADR group

**Figure 7:**
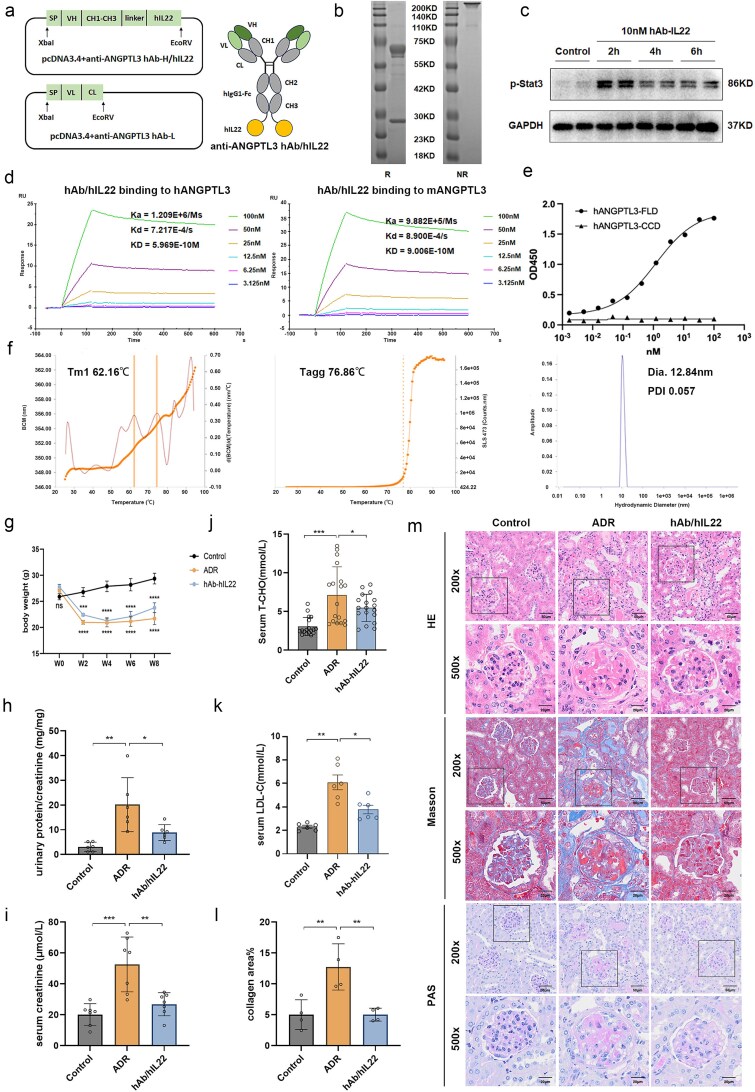
Generation and protection of humanized bifunctional proteins in ADR group mice models; (a) schematic representation of the preparation and purification of the anti-ANGPTL3 hAb/hIL-22 fusion protein; (b) SDS-PAGE analysis of anti-ANGPTL3 hAb/hIL-22 under reducing and non-reduced conditions; anti-ANGPTL3 hAb /hIL-22: ~181 kDa; (c) after mouse proximal tubular epithelial cells (mPTCs) were incubated overnight, the hAb/hIL-22 (10 nM)-induced phosphorylation of STAT3 (p-STAT3) was analyzed using western blotting at different times; (d) affinity of the anti-ANGPTL3 hAb/hIL-22 antibody for mouse and human ANGPTL3 as measured using SPR; (e) ELISA results demonstrate that the binding site was on the C-terminal fibrinogen-like domain rather than the N-terminal coiled-coil domain; (f) by monitoring the changes in particle size in a thermal ramp, the melting temperature (Tm) and aggregation temperature (Tagg) of hAb/hIL-22 were calculated to reflect the unfolding and aggregation characteristics of proteins during the heating process, respectively, corresponding to their thermal stability and colloidal stability; (g, j) body weight and serum T-CHO of mice after hAb/hIL-22 therapy; (h, i, k) measurements of urinary protein/creatinine, serum LDL-C, and serum creatinine levels; (m) representative H&E, Masson, and PAS staining images of kidney samples from ADR mice treated with hAb/hIL-22; (l) ratio of the collagen areas in Masson staining. ^*^P < .05, ^**^P < .01, ^***^P < .001.

To reduce immunogenicity in humans and facilitate clinical translation, the anti-ANGPTL3 antibody was humanized by retaining the CDR sequences and replacing other murine sequences with their human counterparts. The resulting humanized antibody was designated as anti-ANGPTL3 hAb. The bifunctional fusion protein was constructed by fusing human IL-22 to the C-terminus of the heavy chain of the anti-ANGPTL3 hAb via a (Gly4Ser)_3_ linker, as described previously [15]. (Fig. 7a). The recombinant sequence was used for subsequent protein expression to obtain the target protein, denoted as anti-ANGPTL3 hAb/hIL-22.

Various methods and instruments have been used to characterize the anti-ANGPTL3 hAb/hIL-22 fusion proteins. In this study, SDS-PAGE analysis showed distinct bands at the expected molecular weights under both reducing and non-reducing conditions, confirming high protein purity (Fig. 7b). SPR is an established method for detecting antigen–antibody-binding affinity, which reveals the high affinity of the fusion protein for human- or mouse-derived ANGPTL3 (Fig. 7d). The ELISA results further demonstrated that the binding site was on the C-terminal FLD rather than on the N-terminal CCD (Fig. 7e). The thermal and colloidal stabilities were reflected in the Tm1 and Tagg values, respectively, which were detected using an Uncle instrument. The Tm1 value of the fusion protein was 62.16°C, indicating favorable thermal stability (Fig. 7f). The Tagg value of the fusion protein was 76.86°C, suggesting excellent colloidal stability (Fig. 7f). The diameter of the fusion protein was 12.84 nm, and its Polydispersity Index (PDI) was 0.057, implying that it had good homogeneity and narrow size distribution in solution (Fig. 7f). In addition, the biological activity of IL-22 was confirmed via immunoblot analysis, which revealed a significant increase in the phosphorylation of Stat3 after intervention with the fusion protein (Fig. 7c and Supplementary Fig. S3).

The therapeutic effects of the anti-ANGPTL3 hAb/hIL-22 fusion protein were evaluated in ADR group. Compared to the control group, the ADR group showed significant weight loss, increased urinary protein-to-creatinine ratio, elevated serum creatinine, and increased total serum cholesterol and low-density lipoprotein cholesterol levels, indicating that the ADR model was successfully established (Fig. 7g–k). On this basis, the mice in the treatment groups received intraperitoneal injections of the anti-ANGPTL3 hAb/hIL-22 (25 mg/kg) twice a week for 8 weeks. The results revealed that anti-ANGPTL3 hAb/hIL-22 treatment significantly decreased the UACR and serum creatinine as well as serum T-CHO and low-density lipoprotein cholesterol levels, suggesting promising therapeutic effects for alleviating proteinuria, protecting renal function, and regulating dyslipidemia (Fig. 7g–k). In terms of renal histopathology, the model group exhibited severe pathological injury, manifesting as glomerular mesangial expansion and sclerosis, accompanied by tubular dilation and casts, as well as interstitial fibrosis (Fig. 7l and m). Treatment with anti-ANGPTL3 hAb/hIL-22 effectively reversed pathological renal damage and promoted normal tissue repair by alleviating renal fibrosis (Fig. 7l and m). Collectively, these results confirm that we humanized the fusion proteins and retained the bioactivities of the antibodies ANGPTL3 and IL-22, laying the foundation for promoting its clinical translation. The results of further action on the NS mouse model suggest that the humanized fusion proteins could reduce the degree of proteinuria in NS and provide a new therapeutic approach for NS.

## Discussion

Despite rigorous treatment with glucocorticoids, immunosuppressants, and antihypertensive medications to preserve Ccr and renal function, NS remains a major cause of ESRD [17]. Therefore, novel therapeutic strategies targeting NS pathogenesis and preventing its progression to ESRD are urgently required. Since NS progression is strongly associated with elevated ANGPTL3 and reduced IL-22 levels, we employed a presynthesized anti-ANGPTL3/IL-22 bifunctional fusion protein for the treatment of NS.

In our previous study, an anti-ANGPTL3/IL-22 bifunctional fusion protein demonstrated positive therapeutic effects on inflammatory responses and renal fibrosis in a diabetic nephropathy model [15]. However, its role in NS models has not been reported. In this study, we evaluated the therapeutic effects of the fusion protein in ADR group. We found that it effectively protected renal function, attenuated glomerular and tubular injuries, and ameliorated renal fibrosis. Mechanistically, we demonstrated that the fusion protein exerted a renoprotective effect by reducing apoptosis, mitochondrial dysfunction, and autophagy.

Mitochondrial dysfunction occurs early in podocyte injury and is accompanied by altered mitochondrial morphology and increased oxidative stress [18, 19]. Changes in mitochondrial morphology involving continuous fusion and fission are associated with the occurrence and progression of various glomerular diseases [20]. In the ADR group used in the present study, mitochondrial abnormalities were characterized by swelling, disorganization, fragmented cristae, and occasional vacuolar degeneration. Notably, mitochondrial morphology partially returned to normal after treatment with anti-ANGPTL3/IL-22, anti-ANGPTL3, and mIL-22-Fc.

Oxidative stress plays an important role in podocyte injury, which leads to proteinuria, a potential cause of ADR-induced nephrotoxicity [21]. Previous studies have shown that mitochondrial damage increases ROS production, which, in turn, leads to mitochondrial dysfunction, forming a “vicious cycle” related to the occurrence and development of NS [22]. In this study, we focused on two indicators, SOD and MDA, to determine the level of oxidative stress in NS mice. SOD, a critical antioxidant enzyme, catalyzes the dismutation of superoxide radicals into oxygen and hydrogen peroxide, which play key roles in cellular antioxidant defense [23]. SOD levels tend to decrease under oxidative stress. In contrast, MDA is produced by cellular lipid oxidation, and higher levels of MDA indicate greater oxidative stress [24]. Our study revealed that adriamycin induced a decrease in SOD and an increase in MDA levels; these results are supported by earlier studies [25, 26]. This may occur because several oxidoreductases reduce adriamycin by one electron to form ADR-semiquinone free radicals, which, in turn, trigger a series of reactions that produce ROS [27]. Previous findings have suggested that anti-ANGPTL3 antibodies can reduce mitochondrial damage in podocytes through changes in mitochondrial morphology and function aside from decreasing ROS production [9]. IL-22 has also been shown to attenuate oxidative stress and protect mitochondrial function in oxalate-induced crystalline kidney injury and in a murine model of diabetic nephropathy [28]. Notably, oxidative stress was most effectively inhibited by anti-ANGPTL3/IL-22 combination therapy, surpassing the effects of either anti-ANGPTL3 or mIL-22-Fc monotherapy.

Renal cell apoptosis is a major response to various injuries, including impaired glomerular filtration, hypertension, hyperglycemia, and the action of various cytokines and hormones [29]. In addition to direct damage, ADR-induced oxidative stress plays a pivotal role in initiating apoptosis by generating excessive ROS, which compromises the mitochondrial membrane and activates downstream regulators of mitochondria-dependent apoptotic pathways, including the pro-apoptotic Bax and anti-apoptotic Bcl-2 proteins [30, 31]. This study revealed that adriamycin upregulates Bax and downregulates Bcl-2, leading to the cleavage and activation of caspase-3, a key executor of the apoptotic pathway, ultimately promoting cell death. This process is considered a response to inflammation and oxidative stress by initiating and activating pro-apoptotic factors, consistent with that reported by Wang *et al*. [32]. Notably, the administration of the anti-ANGPTL3/IL-22 bifunctional fusion protein reversed the imbalance in the Bax/Bcl-2 ratio and reduced the protease cleavage cascade induced by activated caspase-3. The anti-apoptotic effects of the fusion protein may stem from its protective properties on the mitochondria and ability to alleviate oxidative stress, consistent with previous reports [33]. In addition, Ji *et al*. [10] and Lv *et al*. [9] reported that anti-ANGPTL3 antibody therapy protects podocytes from ADR-induced apoptosis, which may be related to inhibiting the activation of integrin αvβ3 and Rac1 and alleviating mitochondrial fragmentation and dysfunction to attenuate apoptosis [9, 10]. Moreover, IL-22 has anti-apoptotic effects on both acute kidney injury and diabetic nephropathy [14, 34]. Therefore, fusion or combined application of anti-ANGPTL3 and IL-22 may synergistically protect podocytes from apoptosis.

Autophagy is a dynamic process by which damaged macromolecules and organelles are degraded and recycled for the synthesis of new cellular components [35]. Basal autophagy is essential for the preservation of normal kidney physiology. However, autophagic homeostasis is disrupted under pathological conditions. In the ADR-induced nephropathy model, we found that autophagy was activated after ADR induction, which was supported by immunohistochemical results showing increased LC3-II levels and TEM results showing an increase in intracellular autolysosomes. Similar results have been reported in other studies [10]. Strikingly, the autophagic process was downregulated following treatment with the anti-ANGPTL3/IL-22 fusion protein, anti-ANGPTL3 antibody, and mIL-22-Fc, with the fusion protein showing the strongest effect. Ensuring homeostasis of the autophagy has been demonstrated to protect against renal tubular cell death in kidney injury [36] and podocyte death in lupus nephritis [37] and diabetic nephropathy [38]. Notably, to date, there is no clear evidence that ANGPTL3 is strongly associated with autophagic responses. However, our data suggest that reducing ANGPTL3 signaling attenuates LC3II expression and further decreases the number and volume of autolysosomes, so the relationship between ANGPTL3 signaling and autophagy will be explored in the future studies. In addition, studies have confirmed that, oxidative stress leads to damage to organelles such as mitochondria and lipid peroxidation, thereby promoting autophagy [39]. Moreover, autophagy activated by oxidative stress can induce Bnip3-mediated alterations in mitochondrial membrane permeability (mitochondrial permeability transition) and mitochondrial damage, exacerbating cellular injury [40]. Therefore, autophagy and oxidative stress can form a positive feedback loop, ultimately leading to cellular apoptosis.

Our study still has some limitations. Firstly, to improve the efficacy of anti-ANGPTL3/IL-22 fusion proteins used in this study, it would be better to try to determine the optimal dose and frequency of administration. Secondly, the autophagic mechanisms associated with NS remain underexplored. Focusing on specific autophagic stages and key autophagic pathways will facilitate a deeper understanding of the pathophysiology of NS and potential therapeutic strategies. Thirdly, all experiments from this study were conducted in mice, and translation of results for NS patients is still required. Therefore, trials to test the efficacy and safety of the fusion protein are further needed. At last, the initial design of the bifunctional fusion protein aimed to integrate the dual effects of anti-ANGPTL3 monoclonal antibody in reducing podocyte injury and IL-22 in suppressing inflammatory responses. However, in practical applications, due to targeted molecules’ differences in tissue distribution, expression levels, and affinity to the fusion protein, along with constraints such as the 1:2 stoichiometric ratio of the antibody to IL-22 and spatial conformation limitations, this design is unlikely to be considered an optimal solution. Consequently, it has yet to demonstrate efficacy significantly superior to that of the two individual protein monomers. This study preliminarily validates the feasibility of combining the two for treating kidney disease, and future work will focus on optimizing their structure to achieve better therapeutic outcomes. According to protein engineering research on antibody-cytokine fusion therapeutics, alternative architectures including fusing the cytokine moiety to only one heavy chain of the IgG scaffold and engineered single-chain IL-22 variants are feasible structural optimization strategies for future molecular modification. These optimized constructs may maintain comparable targeted renal protective efficacy via ANGPTL3-mediated tissue localization, and further reduce the systemic cytokine burden to enhance the overall safety profile.

In conclusion, this study highlights the therapeutic potential and underlying mechanisms of an anti-ANGPTL3/IL-22 bifunctional fusion protein in NS. The fusion protein effectively alleviates renal dysfunction and pathological damage by preserving mitochondrial integrity, reducing apoptosis, and downregulating autophagy. Furthermore, humanization efforts have advanced the potential for clinical translation. Transcriptomic analyses provide insights into the molecular basis underlying the superior efficacy of fusion proteins compared to monotherapies.

## Supplementary Material

Supplementary_files_tbag031

## Data Availability

The datasets generated and analyzed during the current study are available from the corresponding author upon reasonable request. Some data may be restricted due to privacy or ethical concerns.

## Animal research statement

All animal experiments were approved by the Ethical Committee of Fudan University (Shanghai, China) (IDM2025023) and conducted in compliance with the National Institutes of Health Guidelines for animal research. This study complies with the ARRIVE guidelines.
