# Supplementary material for: Anti-ANGPTL3 antibody and IL-22 fusion protein mitigate nephrotic syndrome via mitochondrial protection, anti-apoptosis, and autophagy suppression
Source: Antib Ther. 2026 Jun 16;9(3):420–36. doi: 10.1093/abt/tbag031 (PMC13463637; doi:10.1093/abt/tbag031)
Supplement: Supplementary file 1 [file supplementary_files_tbag031.docx]

**Supplementary files**

**Supplementary image 1**

Differentially expressed genes (DEGs) between the IL-22Fc and model groups and DEGs between the normal and model groups were projected on the x-axis and y-axis, respectively, according to their log2FC values. The correlation coefficients and p-values of the DEGs between two groups are indicated.

**Supplementary image 2**

Differentially expressed genes (DEGs) between the anti-ANGPTL3 and model groups and DEGs between the normal and model groups were projected on the x-axis and y-axis, respectively, according to their log2FC values. The correlation coefficients and p-values of the DEGs between two groups are indicated.

**Supplementary image 3**

GAPDH 37kd

P-STAT3 86kd

Repeat 1+2

8H

4H

2H

55kd

70kd

35kd

40kd

35kd

40kd

70kd

100kd

100kd

Repeat 3+4

CONTROL

10nM hAb-IL22

**The original western blot images, obtained in the present study, are uncropped and unedited following the publication ethics policies.**
